# Increased type III interferons and NK cell functions in SARS-CoV-2-infected children

**DOI:** 10.1038/s41392-023-01340-8

**Published:** 2023-02-03

**Authors:** Seong Dong Jeong, Hoyoung Lee, Ju Young Chang, Seong Yong Lee, Ji Eun Choi, Eunmi Yang, Hye Won Jeong, Jae-Phil Choi, Mi Seon Han, Eui-Cheol Shin

**Affiliations:** 1 The Center for Viral Immunology, Korea Virus Research Institute, Institute for Basic Science (IBS), Daejeon, 34126 Republic of Korea; 2 Department of Pediatrics, Seoul Metropolitan Government-Seoul National University Boramae Medical Center and Seoul National University of College of Medicine, Seoul, 07061 Republic of Korea; 3 Division of infectious diseases, Seoul Medical Center, Seoul, 02053 Republic of Korea; 4 Department of Internal Medicine, Chungbuk National University College of Medicine, Cheongju, 28644 Republic of Korea; 5 Graduate School of Medical Science and Engineering, Korea Advanced Institute of Science and Technology (KAIST), Daejeon, 34141 Republic of Korea

**Keywords:** Innate immunity, Adaptive immunity

**Dear Editor**,

Severe acute respiratory syndrome coronavirus 2 (SARS-CoV-2) infection is more likely to progress to severe disease in the elderly, and the rates of hospitalization for coronavirus disease 2019 (COVID-19) increase with age.^1,2^ Herein, we comprehensively compared adaptive and innate immune responses between children and adults infected with SARS-CoV-2.

A total of 57 children and 57 adult patients with confirmed SARS-CoV-2 infection were enrolled in this study. Disease severity was determined according to the NIH severity of illness categories: asymptomatic, mild, moderate, severe, and critical.^3^ In the current study, we grouped patients into three categories: asymptomatic, non-severe (mild and moderate), and severe (severe and critical). Thus, this study included 14 asymptomatic children, 43 non-severe children, 30 non-severe adults, and 27 severe adults, and we compared various immunological parameters among these four groups, particularly between non-severe children and adult groups. Clinical information is presented in Supplementary Table 1.

First, we compared the viral load that measured by real-time reverse-transcription (RT)-PCR of nasal swab specimens at the time of diagnosis and found no significant difference in cycle threshold (Ct) values among the four groups (Supplementary Fig. 1).

Next, we evaluated plasma neutralizing antibody (nAb) activity against SARS-CoV-2 at two different phases: early (days post-symptom onset [DPSO], 1–7 days) and late (DPSO, 8–18 days) phases. In the case of asymptomatic infection, days post-diagnosis (DPD) was used instead of DPSO. In the early phase, the severe adult group exhibited significantly higher nAb titers than the other three groups (*p* = 0.04 or *p* < 0.0001 or *p* = 0.001), and the non-severe adult group had significantly higher nAb titers than the non-severe children group (*p* = 0.0002) (Supplementary Fig. 2a). These differences tended to be maintained in the late phase. In the late phase, the severe adult and non-severe adult groups exhibited significantly higher nAb titers than the non-severe children group (*p* = 0.003 or *p* = 0.01) (Supplementary Fig. 2b). Our nAb data are in line with a previous study showing the highest nAb titer in severe patients.^4^

We also examined SARS-CoV-2-specific T-cell responses by performing intracellular cytokine staining (ICS) assays for interferon-γ (IFN-γ) using peripheral blood mononuclear cells (PBMCs) obtained in the late phase (DPSO, 8-19 days). In ICS assays, we stimulated PBMCs with overlapping peptide (OLP) pools covering the spike protein of SARS-CoV-2. The frequency of IFN-γ^+^ cells among CD4^+^ T cells was higher in the non-severe adult group than in the asymptomatic children and non-severe children groups (Supplementary Fig. 3b). It was also higher in the severe adult group than in the non-severe children group (Supplementary Fig. 3b). In the case of CD8^+^ T cells, there was no significant difference in the frequency of IFN-γ^+^ cells among the four groups (Supplementary Fig. 3c). Thus, SARS-CoV-2-specific T-cell responses tended to be higher in adults than in children.

We investigated plasma levels of type I, II, and III IFNs in the early phase (DPSO, ≤ 9 days). Plasma levels of IFN-α2, -β, and -γ were not different among the four patient groups (Fig. 1a and Supplementary Fig. 4a). Among type III IFNs, plasma levels of IFN-λ1 were significantly higher in the non-severe children group than in the severe adult group (*p* = 0.03) (Fig. 1a). In addition, plasma levels of IFN-λ2/3 were significantly higher in the non-severe children group than in the non-severe and severe adult groups (*p* = 0.01 or *p* < 0.0001) (Fig. 1a). Moreover, plasma levels of IFN-λ2/3 were significantly higher in the asymptomatic children group than in the severe adult group (*p* = 0.007) (Fig. 1a). We also analyzed the correlation between the age of patients and plasma levels of IFNs (Fig. 1b and Supplementary Fig. 4b). Among type I, II, and III IFNs, IFN-λ1 and IFN-λ2/3 exhibited inverse correlations with patient age (Fig. 1b). Of note, there was no significant difference in plasma IFN levels between healthy children and adults (Supplementary Fig. 5).

**Fig. 1:**
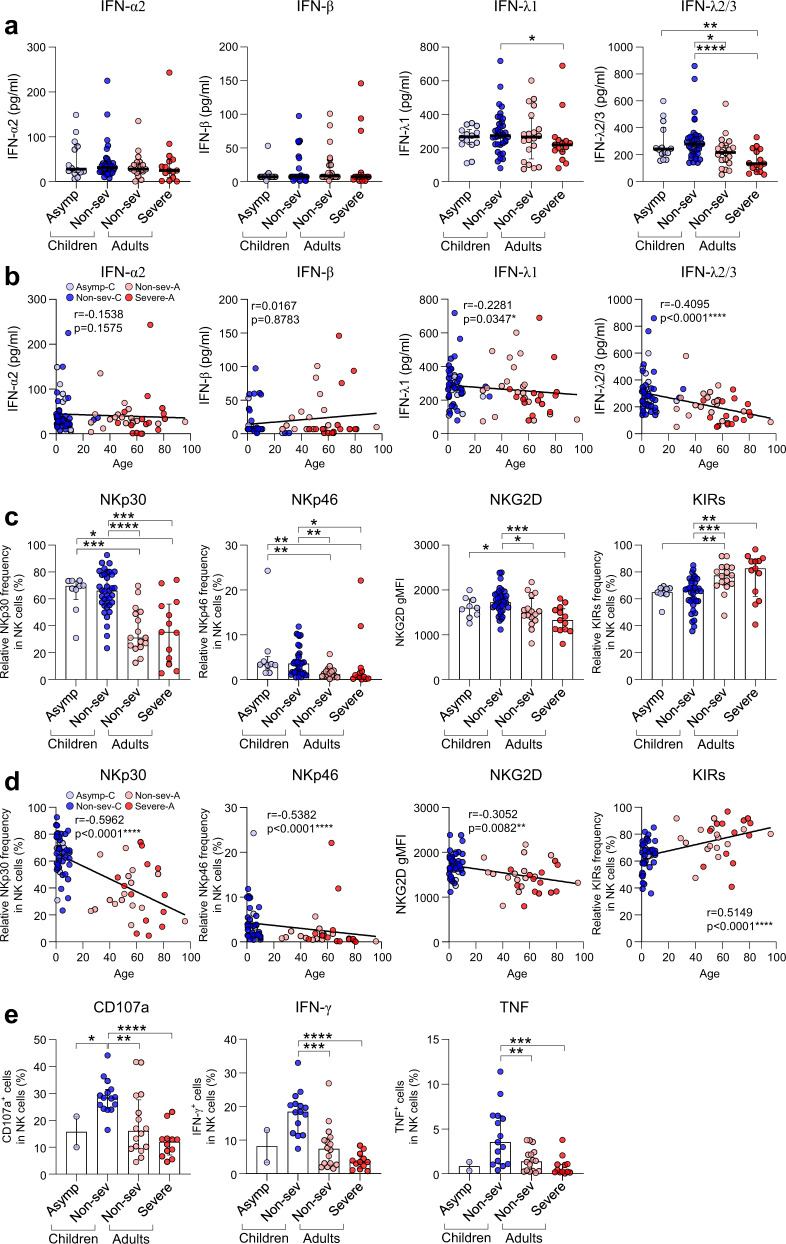
A comparison of immunological profiles after SARS-CoV-2 infection between children and adults. **a** Plasma concentrations of type I (IFN-α2 and IFN-β) and type III (IFN-λ1 and IFN-λ2/3) IFNs were evaluated at an early time point in infection in children (Asymptomatic [Asymp], *n* = 14; Non-severe [Non-sev], n = 37) and adults (Non-severe [Non-sev], *n* = 20; Severe, *n* = 15). **b** Correlation between age and the plasma concentrations of type I and III IFNs. **c** The frequency and expression level of NKp30, NKp46, NKG2D, and KIRs (KIR2D and KIR3DL1/L2) in NK cells. Children (Asymp, *n* = 9; Non-sev, *n* = 36) and adults (Non-sev, *n* = 16; Severe, *n* = 13). **d** Correlation between age and the frequency and expression level of NKp30, NKp46, NKG2D, and KIRs in NK cells. **e** NK cell effector functions against K562 cells. The frequency of CD107a, IFN-γ, and TNF in NK cells was measured after co-culture with K562 cells for 6 h. Children (Asymp, *n* = 2; Non-sev, *n* = 15) and adults (Non-sev, *n* = 16; Severe, *n* = 12). Data are presented as median and interquartile range (IQR). Statistical analysis was performed using the Mann–Whitney *U*-test. *p < 0.05, **p < 0.01, ***p < 0.001, ****p < 0.0001. Spearman correlation test was used to assess the degree of association between two variables. *p < 0.05, **p < 0.01, ****p < 0.0001

Next, we analyzed the phenotypes of CD3^-^CD14^-^CD19^-^CD56^+^ natural killer (NK) cells using PBMC samples from the early phase (DPSO, ≤ 9 days; Supplementary Fig. 6a). In the gate of NK cells, we examined the expression of activating NK receptors and found that the frequency of NKp30^+^ and NKp46^+^ cells among NK cells was significantly higher in the asymptomatic children and non-severe children groups than in the non-severe and severe adult groups (NKp30, *p* = 0.0006 or *p* = 0.01 or *p* < 0.0001 or *p* = 0.0002; NKp46, *p* = 0.001 or *p* = 0.008 or *p* = 0.002 or *p* = 0.01) (Fig. 1c). In addition, the geometric mean fluorescence intensity (gMFI) of NKG2D was significantly higher in the non-severe children group than in the non-severe and severe adult groups (*p* = 0.02 or *p* = 0.0002) (Fig. 1c). It was also significantly higher in the asymptomatic children group than in the severe adult group (*p* = 0.04). Among activating NK receptors, NKG2C presented a different expression pattern. The frequency of NKG2C^+^ cells among NK cells was significantly higher in the non-severe adult group than the asymptomatic children and non-severe children groups (*p* = 0.01 or *p* = 0.003) (Supplementary Fig. 6b). Thus, children tend to exhibit increased expression of activating NK receptors except NKG2C.

We also examined the expression of inhibitory NK receptors and found that the frequency of inhibitory killer-cell immunoglobulin-like receptors (KIRs)^+^ cells among NK cells was significantly higher in the non-severe adult group than the asymptomatic children and non-severe children groups (*p* = 0.001 or *p* = 0.0005) (Fig. 1c). It was also significantly higher in the severe adult group than in the non-severe children group (*p* = 0.005). Of note, there was no significant difference in NK cell phenotypes between healthy children and adults (Supplementary Fig. 7).

In the correlation analysis, the frequency of NKp30^+^ and NKp46^+^ cells and the gMFI of NKG2D exhibited inverse correlations with patient age (Fig. 1d). The frequency of NKG2C^+^ and KIRs^+^ cells significantly correlated with patient age (Fig. 1d and Supplementary Fig. 6c). Therefore, NK cells exhibit distinct phenotypes in SARS-CoV-2-infected children compared to adults: higher expression of activating NK receptors and lower expression of KIRs.

Finally, we examined NK cell functions by performing ICS assays with CD107a degranulation marker staining following co-culture with K562 cells. As results, the non-severe children group exhibited higher NK cell functions than the non-severe and severe adult groups, including CD107a degranulation activity (*p* = 0.005 or *p* < 0.0001) and the production of IFN-γ (*p* = 0.0001 or *p* < 0.0001) and tumor necrosis factor (TNF) (*p* = 0.008 or *p* = 0.0004) (Fig. 1e). Of note, there was no significant difference in NK cell functions between healthy children and adults (Supplementary Fig. 8).

Our current study has several limitations. First, we could not compare severe children vs. severe adults because we could not enroll children with severe COVID-19. Second, we examined plasma and PBMC samples instead of respiratory mucosal samples. Third, we could not determine DPSO in asymptomatic cases.

In summary, we identified increased type III IFN responses and NK cell functions in SARS-CoV-2-infected children compared to adults, explaining why SARS-CoV-2 infection manifests as a milder disease in children than adults.

## Supplementary information

Supplementary materials

## Data Availability

The data that support the findings of this study are available from the corresponding author upon request.
