# Supplementary material for: Increased type III interferons and NK cell functions in SARS-CoV-2-infected children
Source: Signal Transduct Target Ther. 2023 Feb 3;8:54. doi: 10.1038/s41392-023-01340-8 (PMC9896443; doi:10.1038/s41392-023-01340-8)
Supplement: Supplementary file 1 — Supplementary materials [file 41392_2023_1340_MOESM1_ESM.docx]

**Supplementary materials for**

**Increased type III interferons and NK cell functions in SARS-CoV-2-infected children**

Seong Dong Jeong^1^, Hoyoung Lee^1^, Ju Young Chang^2^, Seong Yong Lee^2^, Ji Eun Choi^2^, Eunmi Yang^3^, Hye Won Jeong^4^, Jae-Phil Choi^3#^, Mi Seon Han^2#^, Eui-Cheol Shin^1,5#^

^#^Corresponding author: [dasole@seoulmc.or.kr](mailto:dasole@seoulmc.or.kr) (J.-P.C.); [mshanpid@gmail.com](mailto:mshanpid@gmail.com) (M.S.H.); [ecshin@kaist.ac.kr](mailto:ecshin@kaist.ac.kr) (E.-C.S.)

**This file includes:**

Materials and Methods

Supplementary Figure 1 to 8

Supplementary Table 1

**Materials and Methods**

**Patients and specimens**

In this study, 57 children and 57 adult patients with confirmed SARS-CoV-2 infection between May 2020 and February 2021 were enrolled from Seoul Metropolitan Government-Seoul National University Boramae Medical Center, Seoul Medical Center, and Chungbuk National University Hospital, Republic of Korea. SARS-CoV-2 RNA was detected in the patients’ nasopharyngeal swab specimens by real-time RT-PCR. The Ct values of amplified RdRp, E, or N genes were measured at the time of diagnosis. The disease severity of COVID-19 patients was determined according to the NIH severity of illness categories: asymptomatic, mild, moderate, severe, and critical. The non-severe group comprised mild and moderate patients, and the severe group comprised severe and critical patients. Peripheral blood was collected and centrifuged at 1800 rpm for 10 min to obtain plasma. Plasma was stored at -80℃ until use. PBMCs were isolated by density gradient centrifugation of the peripheral blood sample using Lymphocyte Separation Medium (Corning) and cryopreserved in fetal bovine serum (FBS; Corning, NY, USA) supplemented with 10% (v/v) dimethyl sulfoxide (Sigma-Aldrich, St. Louis, USA) until use.

**SARS-CoV-2** **surrogate virus neutralization test (sVNT)**

To assess SARS-CoV-2 neutralizing antibodies, we employed the Surrogate Virus Neutralization Test Kit (Genscript, Piscataway, NJ). Diluted positive control, negative control, and plasma samples were mixed with diluted HRP-RBD solution at a volume ratio of 1:1 and incubated at 37℃ for 30 min. Incubated control and samples were added to a human angiotensin converting enzyme 2 (ACE2) pre-coated plate and incubated at 37℃ for 15 min. Subsequently, the plate was washed with wash solution and developed with 3,3′,5,5′-tetramethylbenzidine (TMB) solution at room temperature for 15 min. Stop solution was added and the optical density (OD) measured at 450 nm. The inhibition rate was calculated using the following equation:

$$Inhibition rate \left( \% \right)=\left( 1-\frac{OD value of Sample}{OD value of Negative Control} \right)\times100\%$$

A positive cut-off value was set as >30% for SARS-CoV-2 neutralizing antibody detection.

**Flow cytometry**

PBMCs were stained with different fluorochrome-conjugated antibodies for NK cell and T cell analysis at 4℃ for 20 min. The LIVE/DEAD Fixable Dead Cell Stain Kit (Invitrogen) was used to exclude dead cells. After washing, cells were fixed and permeabilized using a Foxp3/Transcription Factor Staining Buffer Set (Invitrogen) and further stained with fluorochrome-conjugated antibodies against intracellular proteins for intracellular staining at 4℃ for 20 min. The stained cells were acquired on a LSR II instrument (BD Biosciences, San Jose, CA, USA) and the data analyzed using FlowJo software (Treestar, Ashland, OR, USA).

**T cell functional assay**

PBMCs were cultured in 96-well U-bottom plates at 1×10^6^ cells per well and stimulated with overlapping peptide (OLP) pools covering the whole protein sequence of the SARS-CoV-2 spike protein, PepTivator SARS-CoV-2 Prot_S Complete (Miltenyi Biotec), at a final concentration of 1 µg/ml. After 1 h incubation at 37℃, Brefeldin A (GolgiPlug, BD Biosciences) and monensin (GolgiStop, BD Biosciences) were added. After an additional 5 h incubation at 37℃, cells were harvested and analyzed by flow cytometry.

**Plasma IFN concentration measurement**

The concentrations of IFNs in plasma were measured by LEGENDplex Human Type 1/2/3 Interferon Panel (5-plex: IFN-α2, IFN-β, IFN-λ1, IFN-λ2/3, and IFN-γ) (BioLegend, San Diego, USA) following the manufacturer’s instructions.

**NK cell functional assay**

PBMCs were co-cultured in 96-well U-bottom plates with K562 cells at an effector to target ratio of 5:1 in the presence of anti-CD107a. After 1 h incubation at 37℃, Brefeldin A (GolgiPlug, BD Biosciences) and monensin (GolgiStop, BD Biosciences) were added. After an additional 5 h incubation at 37℃, cells were harvested and analyzed by flow cytometry.

**Statistical analysis**

Statistical analyses were performed using Prism software (GraphPad Software, San Diego, CA, USA). Significance was set at p < 0.05. The Mann-Whitney U test was conducted to compare two unpaired groups of data. Correlations were assessed using the Spearman correlation test.

**
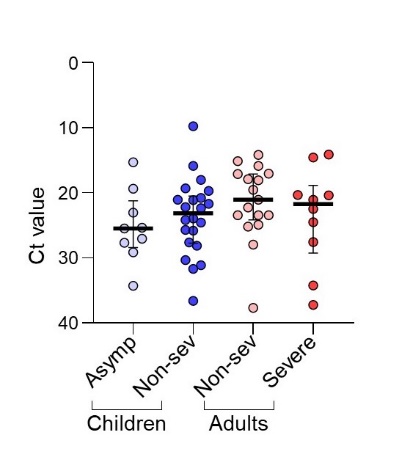
**

**Supplementary Figure 1.** SARS-CoV-2 viral loads at diagnosis were measured by real-time RT-PCR in children (Asymptomatic [Asymp], n=9; Non-severe [Non-sev], n=22) and adults (Non-severe [Non-sev], n=17; Severe, n=10) and represented as cycle threshold (Ct) values. Data are presented as median and interquartile range (IQR). Statistical analysis was performed using the Mann-Whitney U test.

**
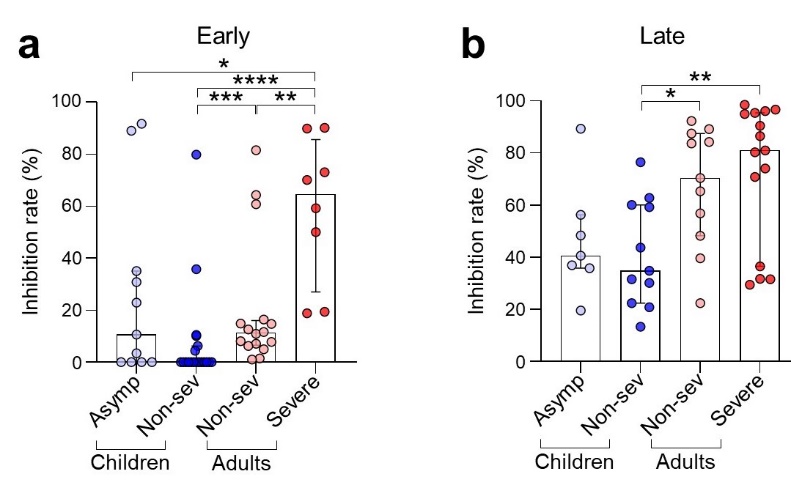
**

**Supplementary Figure 2.** Neutralizing activity against SARS-CoV-2 was evaluated using a surrogate virus neutralization test kit. A positive cutoff value was set at 30% for SARS-CoV-2 neutralizing antibody detection. Children (Asymp, n=11; Non-sev, n=19) and adults (Non-sev, n=16; Severe, n=8) for early time points (DPSO, 1-7 days) and children (Asymp, n=7; Non-sev, n=11) and adults (Non-sev, n=11; Severe, n=15) for late time points (DPSO, 8-18 days). Data are presented as median and IQR. Statistical analysis was performed using the Mann-Whitney U test. **p* < 0.05, ***p* < 0.01, ****p* < 0.001, *****p* < 0.0001.

**
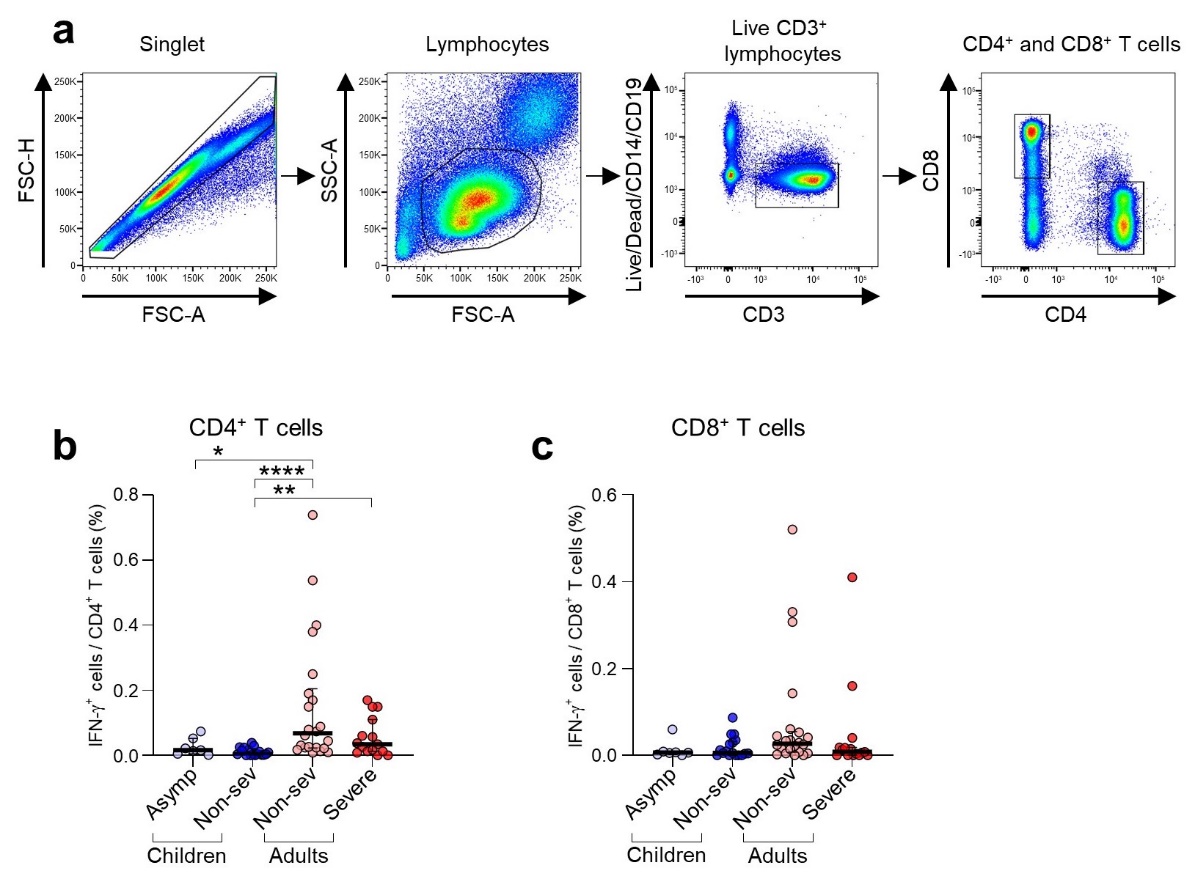
**

**Supplementary Figure 3. T-cell responses against SARS-CoV-2.** (a) Gating strategy and representative flow cytometry plots for T cells. (b and c) The frequency of IFN-γ-producing cells stimulated with SARS-CoV-2 spike protein overlapping peptide (OLP) pools among CD4^+^ and CD8^+^ T cells. Children (Asymp, n=7; Non-sev, n=17) and adults (Non-sev, n=22; Severe, n=15). Data are presented as median and IQR. Statistical analysis was performed using the Mann-Whitney U test. **p* < 0.05, ***p* < 0.01, *****p* < 0.0001.

**
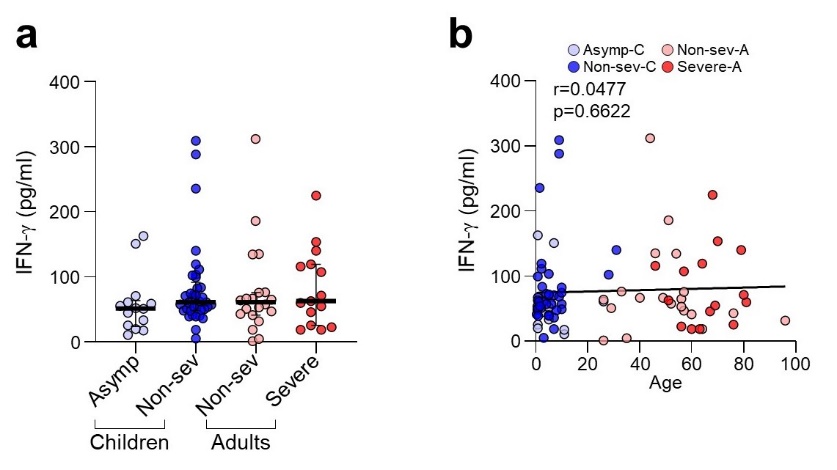
**

**Supplementary Figure 4. Plasma levels of type II IFN in COVID-19 patients.** (a) Plasma concentration of type II (IFN-γ) IFN was evaluated at an early time point in infection in children (Asymp, n=14; Non-sev, n=37) and adults (Non-sev, n=20; Severe, n=15). Data are presented as median and IQR. Statistical analysis was performed using the Mann-Whitney U test. (b) Correlation between age and the plasma concentration of type II IFN. Spearman correlation test was used to assess the degree of association between two variables.

**
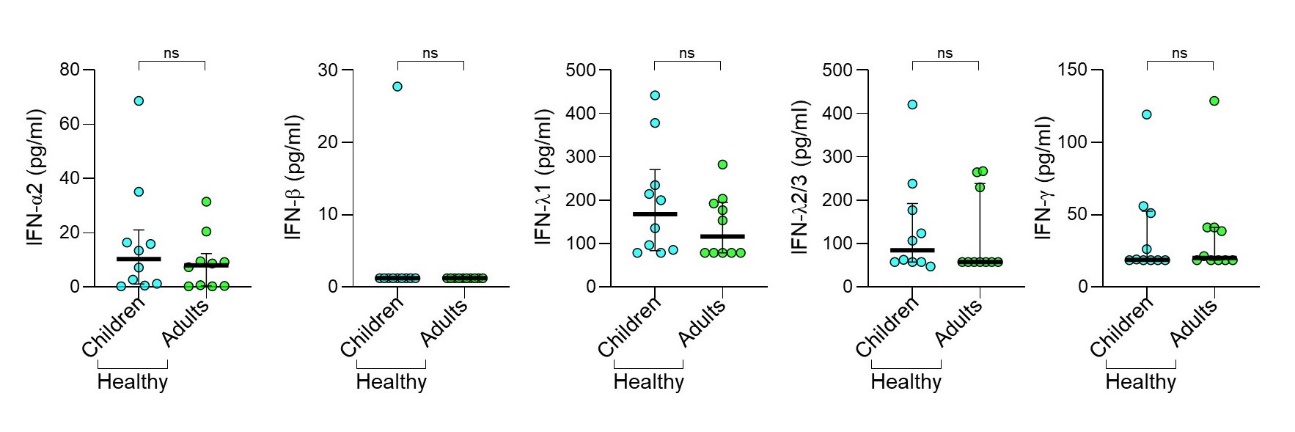
**

**Supplementary Figure 5.** Plasma concentrations of type I (IFN-α2 and IFN-β), type II (IFN-γ), and type III (IFN-λ1 and IFN-λ2/3) IFNs were evaluated in healthy children (n=10) and adults (n=10). Data are presented as median and IQR. Statistical analysis was performed using the Mann-Whitney U test. ns, nonsignificant.

**
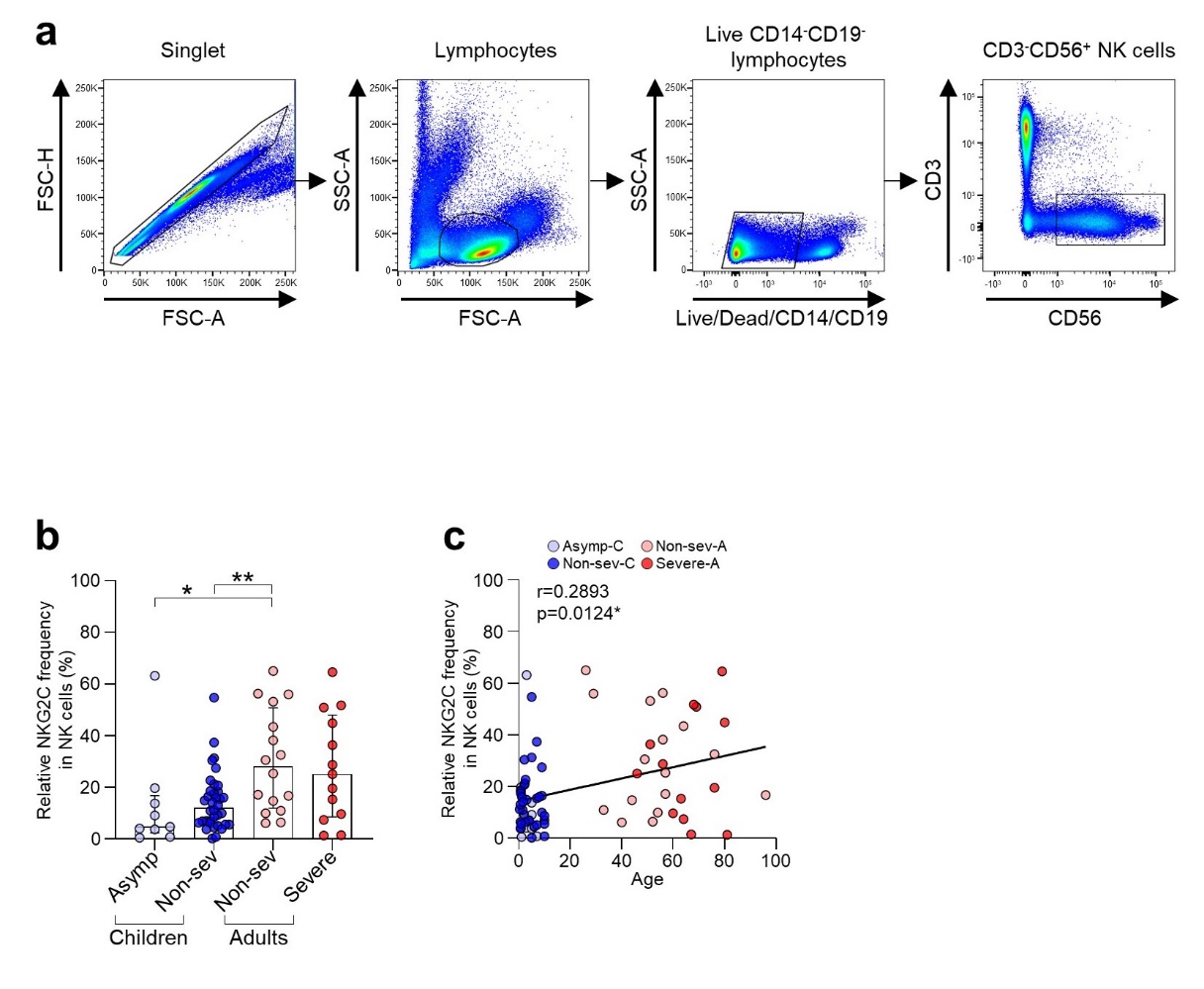
**

**Supplementary Figure 6. Phenotypical changes in NK cells in COVID-19.** (a) Representative flow cytometry plots showing the gating strategy for CD3^-^CD56^+^ NK cells. (b) The frequency of NKG2C in NK cells. Children (Asymp, n=9; Non-sev, n=36) and adults (Non-sev, n=16; Severe, n=13). Data are presented as median and IQR. Statistical analysis was performed using the Mann-Whitney U test. **p* < 0.05, ***p* < 0.01. (c) Correlation between age and the frequency of NKG2C in NK cells. Spearman correlation test was used to assess the degree of association between two variables. **p* < 0.05.

**
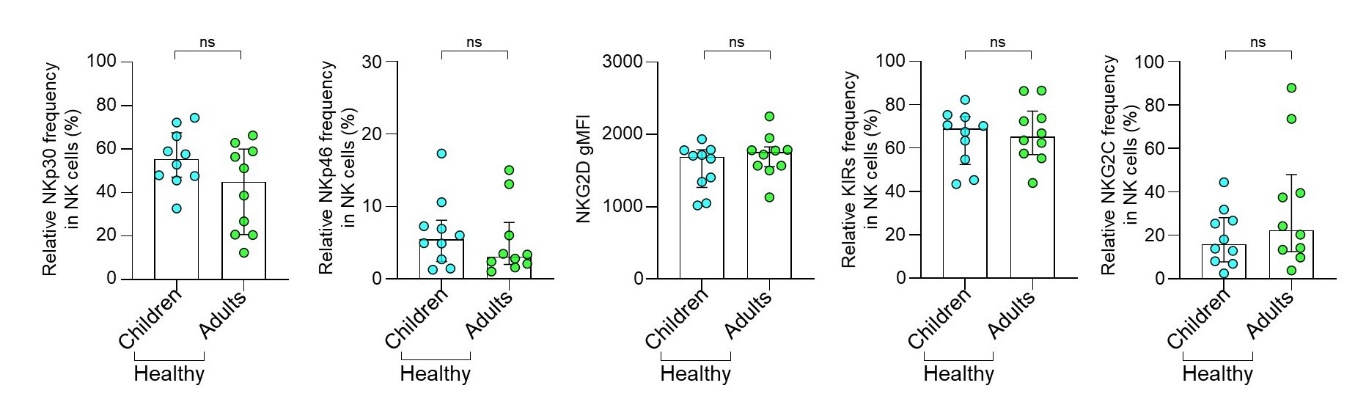
**

**Supplementary Figure 7.** The frequency and expression level of NKp30, NKp46, NKG2D, KIRs (KIR2D and KIR3DL1/L2), and NKG2C in NK cells of healthy children (n=10) and adults (n=10). Data are presented as median and IQR. Statistical analysis was performed using the Mann-Whitney U test. ns, nonsignificant.

**
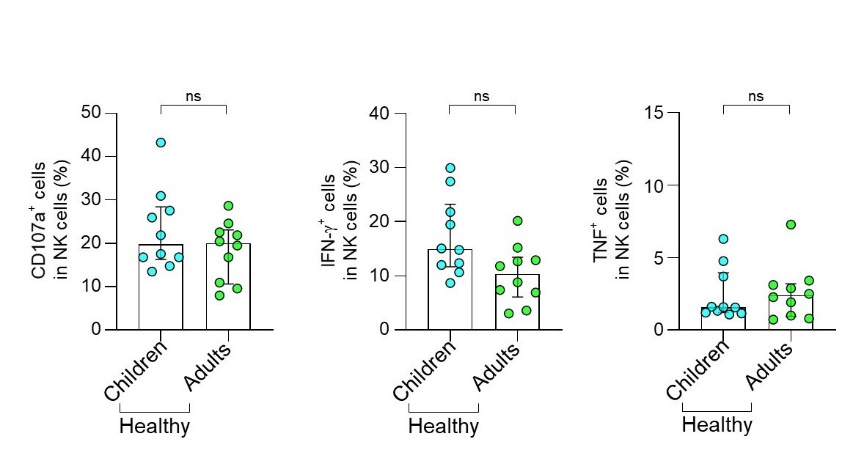
**

**Supplementary Figure 8.** NK cell effector functions against K562 cells. The frequency of CD107a, IFN-γ, and TNF in NK cells was measured after co-culture with K562 cells for 6h in healthy children (n=10) and adults (n=10). Data are presented as median and IQR. Statistical analysis was performed using the Mann-Whitney U test. ns, nonsignificant.

**Supplementary Table 1.** **Characteristics of enrolled patients.** Disease severity of COVID-19 patients was determined according to NIH severity of illness categories: asymptomatic, mild, moderate, severe, or critical. In the case of asymptomatic, the days post-diagnosis (DPD) was used instead of DPSO. M, male; F, female; d, determined; L, late time point of DPSO in each patient.

| **No.** | **Gender** | **Age(yr)** | **Disease severity** | **DPSO** | **Ct value** | **Neutralizing activity** | **T cell response** | **Type I/II/III IFN** | **NK cell phenotype** | **NK cell function** |
| --- | --- | --- | --- | --- | --- | --- | --- | --- | --- | --- |
| C01 | M | 3 | asymptomatic | 5, 9 | d | d, d(L) | d | d | d |  |
| C02 | F | 0.75 | asymptomatic | 1, 8 | d | d, d(L) | d | d | d |  |
| C03 | F | 7 | asymptomatic | 4, 9 | d | d, d(L) | d | d | d |  |
| C04 | F | 1.08 | asymptomatic | 5, 9 | d | d, d(L) | d | d | d |  |
| C05 | M | 3 | asymptomatic | 2, 9 | d | d, d(L) | d | d | d |  |
| C06 | F | 0.83 | asymptomatic | 1 | d | d |  | d |  |  |
| C07 | F | 11 | asymptomatic | 6 | d | d |  | d |  |  |
| C08 | M | 11 | asymptomatic | 5 | d | d |  | d |  |  |
| C09 | M | 0.83 | asymptomatic | 2 | d | d |  | d |  |  |
| C10 | M | 5 | asymptomatic | 1, 9 |  | d, d(L) | d | d | d |  |
| C11 | M | 6 | asymptomatic | 1, 9 |  | d, d(L) | d | d | d |  |
| C12 | M | 5 | asymptomatic | 5 |  |  |  | d | d | d |
| C13 | F | 5 | asymptomatic | 6 |  |  |  | d | d | d |
| C14 | M | 2.16 | asymptomatic | 6 |  |  |  | d |  |  |
| C15 | M | 10 | mild | 3, 9 | d | d, d(L) | d | d | d |  |
| C16 | M | 9 | mild | 6, 12 | d | d | d | d | d |  |
| C17 | M | 3 | mild | 4, 9 | d | d | d | d | d |  |
| C18 | M | 4 | mild | 5 | d | d |  | d | d |  |
| C19 | F | 6 | mild | 5 | d | d |  | d | d |  |
| C20 | F | 0.83 | mild | 3 | d | d |  | d | d |  |
| C21 | M | 0.58 | mild | 7 | d | d |  | d | d |  |
| C22 | M | 2 | mild | 6 | d | d |  | d | d |  |
| C23 | F | 9 | mild | 1 | d | d |  | d | d |  |
| C24 | M | 7 | mild | 2 | d | d |  | d | d |  |
| C25 | F | 1.5 | mild | 5 | d | d |  | d | d |  |
| C26 | M | 2.25 | mild | 6, 9 | d |  | d | d | d | d |
| C27 | F | 0.33 | mild | 6, 9 | d |  | d | d | d | d |
| C28 | M | 3 | mild | 8 |  | d | d | d |  |  |
| C29 | F | 2.58 | mild | 9 |  | d | d |  |  |  |
| C30 | M | 1.33 | mild | 9 |  | d | d |  |  |  |
| C31 | M | 0.08 | mild | 8 |  | d | d |  |  |  |
| C32 | M | 10 | mild | 8 |  | d | d |  |  |  |
| C33 | F | 4 | mild | 3 |  | d |  | d | d |  |
| C34 | M | 5 | mild | 2 |  | d |  | d | d |  |
| C35 | F | 5 | mild | 2, 9 |  |  | d(L) | d | d | d |
| C36 | F | 11 | mild | 10 |  |  | d |  |  |  |
| C37 | M | 0.75 | mild | 2 |  |  |  | d | d | d |
| C38 | M | 5 | mild | 4 |  |  |  | d | d | d |
| C39 | F | 2.5 | mild | 2 |  |  |  | d | d | d |
| C40 | F | 2.25 | mild | 1 |  |  |  | d | d | d |
| C41 | F | 0.58 | mild | 2 |  |  |  | d | d | d |
| C42 | F | 9 | mild | 2 |  |  |  | d | d | d |
| C43 | M | 7 | mild | 2 |  |  |  | d | d | d |
| C44 | M | 2.33 | mild | 2 |  |  |  | d | d | d |
| C45 | F | 2.58 | mild | 2 |  |  |  | d | d | d |
| C46 | F | 0.58 | mild | 2 |  |  |  | d | d | d |
| C47 | M | 7 | mild | 3 |  |  |  |  | d | d |
| C48 | F | 0.75 | moderate | 5, 10 | d | d, d(L) | d | d | d |  |
| C49 | M | 0.75 | moderate | 3, 9 | d | d, d(L) | d | d | d |  |
| C50 | M | 10 | moderate | 8, 17 | d | d | d | d | d |  |
| C51 | M | 10 | moderate | 8, 17 | d | d | d | d | d |  |
| C52 | F | 5 | moderate | 8 | d | d | d | d |  |  |
| C53 | M | 1.16 | moderate | 3 | d | d |  | d | d |  |
| C54 | F | 10 | moderate | 4 | d | d |  | d | d |  |
| C55 | F | 8 | moderate | 3 | d | d |  | d | d |  |
| C56 | M | 0.91 | moderate | 5 | d | d |  | d | d |  |
| C57 | M | 1.58 | moderate | 6 |  |  |  | d | d | d |
| A01 | F | 56 | mild | 4, 11 | d | d | d | d | d | d |
| A02 | M | 33 | mild | 6, 14 | d | d | d | d | d | d |
| A03 | M | 40 | mild | 5, 15 | d | d | d | d | d | d |
| A04 | F | 52 | mild | 7, 19 | d | d | d | d | d | d |
| A05 | M | 46 | mild | 1, 8 | d | d | d | d |  |  |
| A06 | M | 26 | mild | 6, 16 | d | d | d | d |  |  |
| A07 | F | 29 | mild | 7 | d | d |  | d | d | d |
| A08 | M | 26 | mild | 6 | d | d |  | d | d | d |
| A09 | F | 35 | mild | 9 |  |  | d | d |  |  |
| A10 | F | 96 | moderate | 2, 10 | d | d, d(L) | d | d | d | d |
| A11 | F | 49 | moderate | 2, 9 | d | d, d(L) | d | d | d | d |
| A12 | M | 60 | moderate | 9 | d | d | d | d |  |  |
| A13 | F | 44 | moderate | 7 | d | d |  | d | d | d |
| A14 | F | 76 | moderate | 6 | d | d |  | d | d | d |
| A15 | F | 51 | moderate | 5 | d | d |  | d | d | d |
| A16 | M | 54 | moderate | 6 | d | d |  | d | d | d |
| A17 | F | 57 | moderate | 3 | d | d |  | d | d | d |
| A18 | F | 56 | moderate | 5 | d | d |  | d | d | d |
| A19 | M | 38 | moderate | 12 |  | d | d |  |  |  |
| A20 | F | 58 | moderate | 18 |  | d | d |  |  |  |
| A21 | M | 67 | moderate | 10 |  | d | d |  |  |  |
| A22 | F | 51 | moderate | 12 |  | d | d |  |  |  |
| A23 | M | 38 | moderate | 10 |  | d | d |  |  |  |
| A24 | M | 35 | moderate | 14 |  | d | d |  |  |  |
| A25 | M | 61 | moderate | 13 |  | d | d |  |  |  |
| A26 | M | 40 | moderate | 10 |  | d | d |  |  |  |
| A27 | M | 57 | moderate | 7, 14 |  |  | d(L) | d | d | d |
| A28 | F | 64 | moderate | 8, 14 |  |  | d(L) | d | d | d |
| A29 | M | 53 | moderate | 15 |  |  | d |  |  |  |
| A30 | F | 58 | moderate | 19 |  |  | d |  |  |  |
| A31 | F | 67 | severe | 8 | d | d | d | d | d | d |
| A32 | M | 51 | severe | 9 | d | d | d | d | d | d |
| A33 | M | 76 | severe | 6 | d | d |  | d | d | d |
| A34 | F | 69 | severe | 6 | d | d |  | d | d | d |
| A35 | F | 79 | severe | 7 | d | d |  | d | d | d |
| A36 | M | 56 | severe | 7 | d | d |  | d | d | d |
| A37 | M | 80 | severe | 5 | d | d |  | d | d |  |
| A38 | M | 70 | severe | 3, 10 |  | d (L) | d | d |  |  |
| A39 | M | 70 | severe | 14 |  | d | d |  |  |  |
| A40 | M | 60 | severe | 12 |  | d | d |  |  |  |
| A41 | M | 42 | severe | 14 |  | d | d |  |  |  |
| A42 | F | 35 | severe | 12 |  | d | d |  |  |  |
| A43 | M | 74 | severe | 18 |  | d | d |  |  |  |
| A44 | M | 66 | severe | 13 |  | d | d |  |  |  |
| A45 | F | 40 | severe | 13 |  | d | d |  |  |  |
| A46 | F | 66 | severe | 12 |  | d | d |  |  |  |
| A47 | F | 54 | severe | 12 |  | d | d |  |  |  |
| A48 | F | 60 | severe | 1 |  |  |  | d | d | d |
| A49 | M | 63 | severe | 7 |  |  |  | d | d | d |
| A50 | M | 81 | critical | 3 | d | d |  | d | d | d |
| A51 | F | 46 | critical | 7 | d | d |  | d | d | d |
| A52 | F | 68 | critical | 4 | d | d |  | d | d | d |
| A53 | M | 74 | critical | 11 |  | d | d |  |  |  |
| A54 | M | 50 | critical | 13 |  | d | d |  |  |  |
| A55 | F | 62 | critical | 11 |  | d | d |  |  |  |
| A56 | M | 64 | critical | 7 |  |  |  | d | d | d |
| A57 | M | 57 | critical | 8 |  |  |  | d |  |  |
